# Supplementary material for: Antichiral surface states in time-reversal-invariant photonic semimetals
Source: Nat Commun. 2023 Apr 11;14:2027. doi: 10.1038/s41467-023-37670-y (PMC10090124; doi:10.1038/s41467-023-37670-y)
Supplement: Supplementary file 1 — Supplementary Information [file 41467_2023_37670_MOESM1_ESM.pdf]

Supplementary Information for "Antichiral surface states in time-reversal-invariant photonic semimetals"

Jian-Wei Liu[1], Fu-Long Shi[1], Ke Shen[1], Xiao-Dong Chen[1],
Wen-Jie Chen[1*], and Jian-Wen Dong[1*]

[1] *School of Physics & State Key Laboratory of Optoelectronic Materials and Technologies, Sun Yat-sen University, Guangzhou 510275, China.*

[*] Corresponding author: chenwenj5@mail.sysu.edu.cn; dongjwen@mail.sysu.edu.cn

Supplementary Note 1: Tight-binding analysis of AA-stack honeycomb lattice

The unit cell of AA-stack honeycomb lattice is shown in Fig. 1. Corresponding Hamiltonian is expressed as

$$H=\sum_{<i,j>;m}(t_1 a_{i,m}^{\dagger} b_{j,m}+h.c.)+\sum_{<<i,j>>;m}(t_2 a_{i,m}^{\dagger} a_{j,m+1}+t_2 b_{i,m}^{\dagger} b_{j,m+1}+h.c.),$$

where a (b) and $a^{\dagger}$ ($b^{\dagger}$) are the annihilation and creation operators on sublattice sites, i and j label the position of lattice in each layer, m is the layer index. For simplicity, the on-site energy difference between sublattices is neglected. Only the intralayer coupling t_1 [white stick in Fig. 1b] and interlayer coupling t_2 [yellow stick in Fig. 1b] are considered and are both real numbers as restricted by time reversal. By performing the Fourier transform, the lattice Hamiltonian can be rewritten in momentum space, i.e. $H=\sum_{\mathbf{k}}\psi_{\mathbf{k}}^{\dagger}h(\mathbf{k})\psi_{\mathbf{k}}$, where $\psi_{\mathbf{k}}=\left(a_{\mathbf{k}},b_{\mathbf{k}}\right)^{T}$. Therefore, the Bloch Hamiltonian has the following form:

$$H(\mathbf{k})=\begin{pmatrix}\lambda_{\mathbf{k}} & t_1\beta_{\mathbf{k}}\\ t_1\beta_{\mathbf{k}}^{*} & \lambda_{\mathbf{k}}\end{pmatrix},$$

where $\beta_{\mathbf{k}}=1+\exp(ik_x a)+\exp(ik_x a/2+i\sqrt{3}k_y a/2)$, a is the in-plane lattice constant and

$$\begin{aligned}\lambda_{\mathbf{k}}&=t_2\cos k_z d\left[\cos k_x a+\cos\left(k_x a/2+\sqrt{3}k_y a/2\right)+\cos\left(k_x a/2-\sqrt{3}k_y a/2\right)\right]\\&+t_2\sin k_z d\left[\sin k_x a-\sin\left(k_x a/2+\sqrt{3}k_y a/2\right)-\sin\left(k_x a/2-\sqrt{3}k_y a/2\right)\right]\end{aligned}$$

represents the energy modulation by interlayer couplings and k_z, and d is the interlayer distance. The first Brillouin zone (BZ) of this 3D lattice is shown in Fig. 1c.

Effective Modified Haldane model

Via dimension reduction, the non-reciprocal next-nearest-neighbor coupling in the MHM is mimicked by the interlayer coupling in our reduced 2D system with nonzero

k_z. The eigen-spectra with different k_z are shown in Fig. S1. When $k_z = 0$, our system reduces to the case of graphene with identical Dirac frequencies at K and K' points. Yet for the cases with nonzero k_z, the two Dirac points split in energy as the synthetic gauge flux turns on. Then, a tilted edge band pinned at two Dirac points is expected. Notably, because time-reversal symmetry is preserved in our 3D system, the 2D subsystem with positive k_z is the time-reversal counterpart of the one with negative k_z. Fig. S2 shows the eigen-spectral with negative k_z.

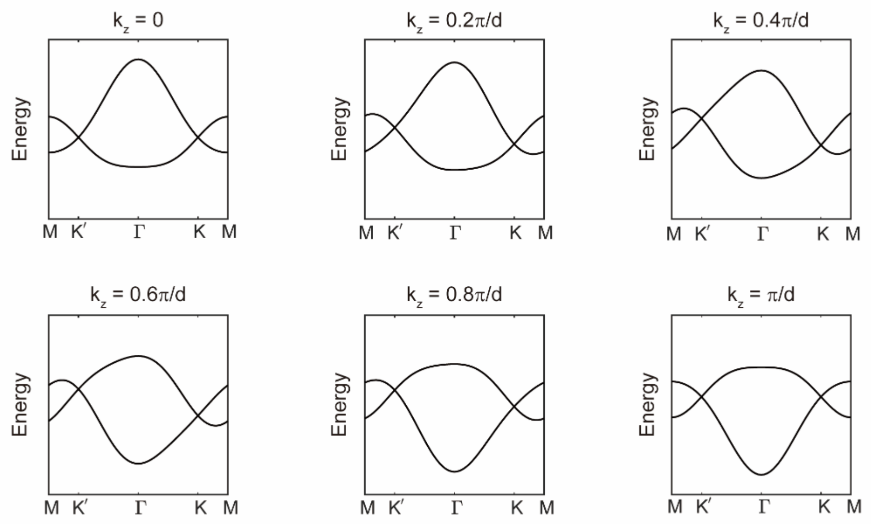

Fig. S1 Eigen-spectra of the Bloch Hamiltonian with positive k_z. Here, the coupling coefficients are set as $t_1 = 1$ and $t_2 = 0.3$.

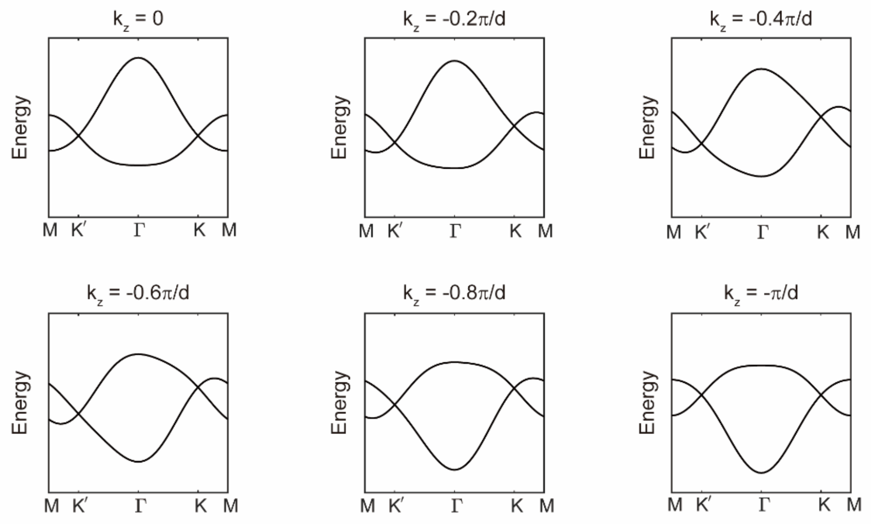

Fig. S2 Eigen-spectra of the Bloch Hamiltonian with negative k_z.

Asymmetric Nodal lines at K and K' valley

The eigen-energy in our system is given by $E(\mathbf{k}) = \lambda_{\mathbf{k}} \pm t_1 |\beta_{\mathbf{k}}|$. At $\overline{\mathrm{K}}$ and $\overline{\mathrm{K}'}$ points, $\beta_{\mathbf{k}} = 0$ and two nodal lines form. Energy at $\overline{\mathrm{K}}$ and $\overline{\mathrm{K}'}$ points can be expressed as

$$E(\overline{\mathrm{K}}) = -\frac{3}{2} t_2 \cos(k_z d) - \frac{3\sqrt{3}}{2} t_2 \sin(k_z d),$$

$$E(\overline{\mathrm{K}'}) = -\frac{3}{2} t_2 \cos(k_z d) + \frac{3\sqrt{3}}{2} t_2 \sin(k_z d).$$

The k_z dispersions of two nodal lines are plotted in Fig. S3. It can be seen that the two nodal lines disperse asymmetrically along k_z. It is this asymmetric dispersion makes the existence of antichiral edge states.

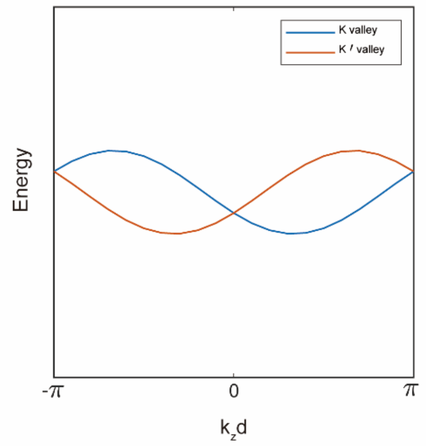

Fig. S3 Asymmetric k_z dispersions of two Dirac nodal lines.

Twisted ribbon surface state

Guaranteed by the opposite Berry phases carried by the two asymmetrically dispersed nodal lines, a nontrivial surface band must fill inside or outside two projected nodal lines. In this subsection, we calculate the surface dispersion onto the projected k_x-k_z plane using tight-binding model. It should be emphasized that the surface band in our system is periodically extended in k_z direction, but resides in a finite k_x regime (pinned at the nodal lines). Besides, with the change of k_z, the nodal lines asymmetrically evolve in energy/frequency, making this surface band twisted in the x direction. Therefore, we describe this surface band structure as a twisted ribbon shape. The twisted ribbon surface state calculated by the tight-binding model is shown in Fig. S4, along with two asymmetric nodal lines (red and blue). Surface modes for certain values of k_z are highlighted with green solid lines. Obviously, surface mode with $k_z = -\pi/2d$ has a

positive group velocity in the x direction, whereas the one with $k_z = \pi/2d$ has a negative group velocity.

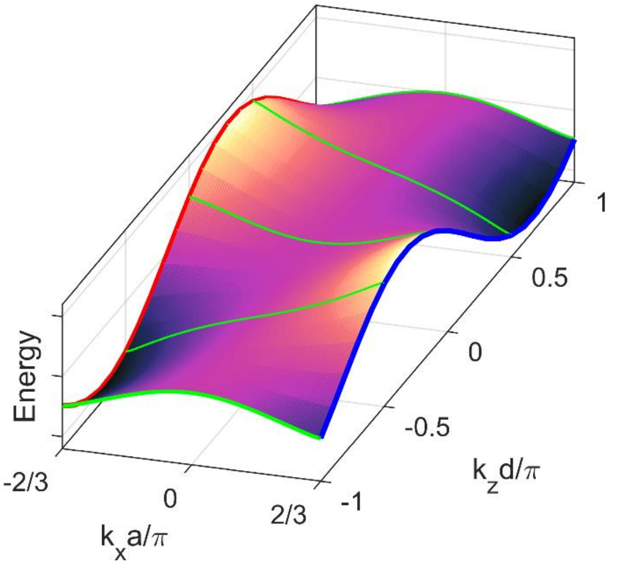

Fig. S4 Twisted ribbon surface dispersion calculated by the tight-binding model. The two asymmetric nodal lines are depicted in red and blue, respectively.

Supplementary Note 2: Eigen mode profiles for structures with/without mirror symmetry

Due to the lack of mirror symmetry σ_z in our system, the eigen modes cannot be simply classified as TE or TM polarization. In this section, we show the evolution of the eigen mode profiles from the case with preserved σ_z to the one with broken σ_z. Naturally, we fix k_z momentum to zero below.

The eigen mode profiles can be classified into TM or TE polarization for structure with mirror symmetry σ_z. As shown in Fig. S4, the unit cell possesses mirror symmetry when the two sets of arms have equal length ($l_1 = l_2 = 0.46a$). Consequently, the first and fourth bands are classified as TM polarization and the second and third bands are classified as TE polarization.

However, this is not the case when $l_1 \neq l_2$. When the mirror symmetry is slightly broken ($l_1 = 0.46a, l_2 = 0.40a$), the TM and TE polarizations begin to couple together and the eigen field becomes the combination of the TM and TE components (see Fig. S5). Further, the coupling between the TM and TE components will become stronger with one set of metallic arms absent (see Fig. S6, corresponding to the case in main manuscript).

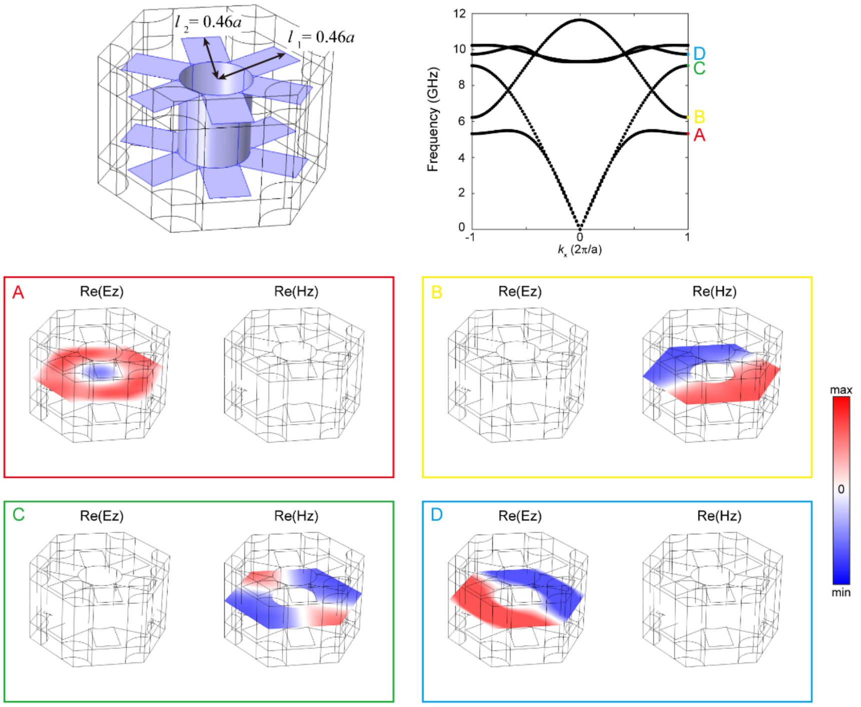

Fig. S4 Photonic metacrystal with mirror symmetry ($l_1 = 0.46a, l_2 = 0.46a$). The first and fourth bands belong to the TM polarization while the second and third bands belong to the TE polarization.

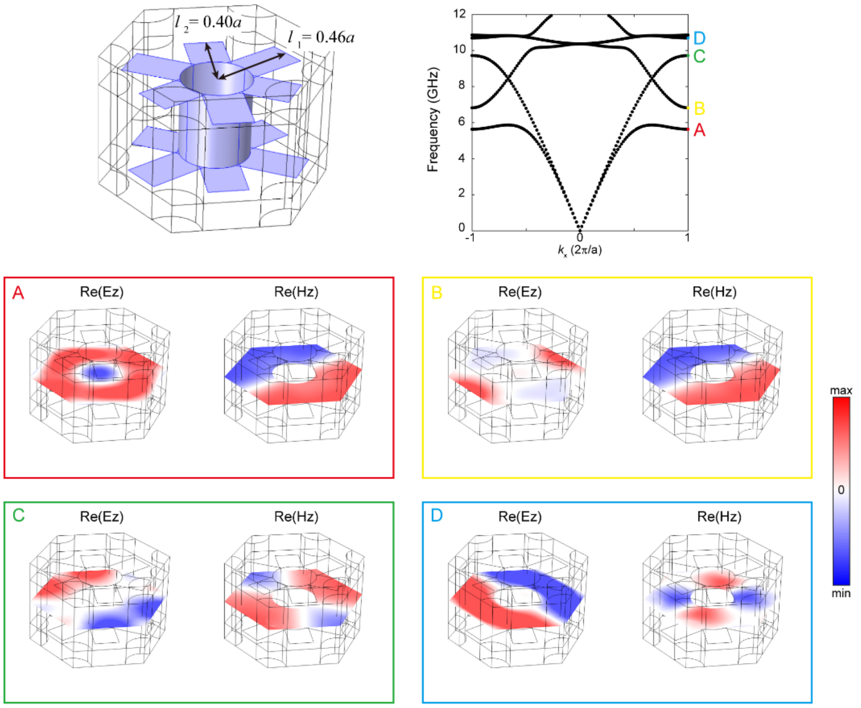

Fig. S5 Photonic metacrystal without mirror symmetry ($l_1 = 0.46a, l_2 = 0.40a$). The TM and TE polarizations begin to couple together and cannot be distinguished.

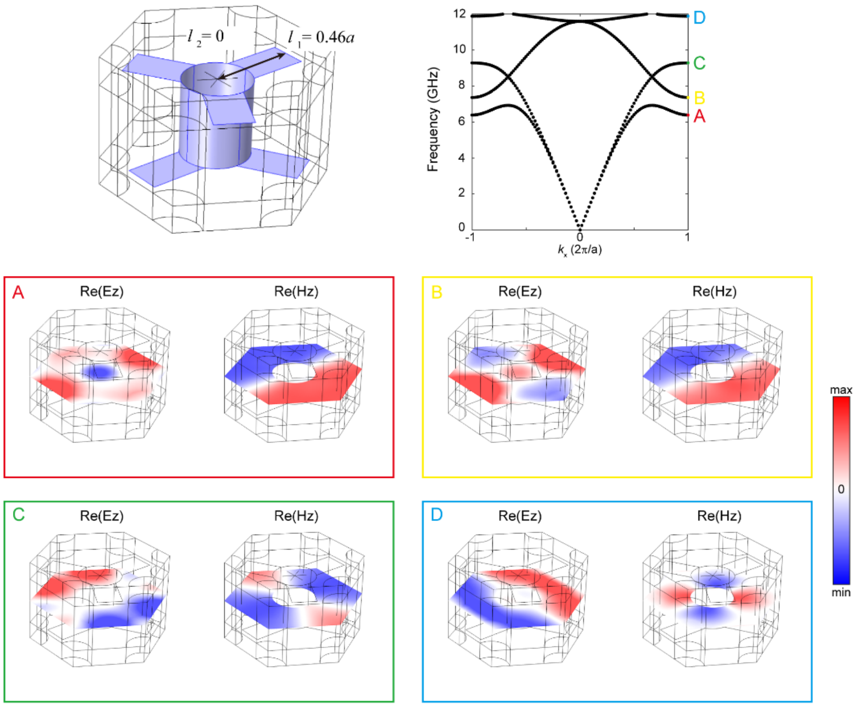

Fig. S6 Photonic metacrystal without mirror symmetry ($l_1 = 0.46a, l_2 = 0$, as discussed in main manuscript).

Supplementary Note 3: Zak phase distribution in the surface Brillouin zone (k_x-k_z plane)

To investigate the topological feature of two ANLs in our metacrystal, we calculate the Zak phases of bulk states. We will show that the existence of antichiral surface state on the *x-z* plane is related to the bulk Zak phase. For each *k*-point in the surface BZ (k_x, k_z), our 3D photonic metacrystal can be deemed as a 1D system along the k_y direction. Consequently, the bulk topology of such 1D subsystem can be described by Zak phase. Figure S7a shows the band structure on the plane of $k_z = 0.2\pi/d$. In the k_x direction, the second and third bands intersect and form two Dirac points. In the k_y direction, the lowest three bands are isolated. We calculate the Zak phases of band 1 and band 2 as θ_1 and θ_2 (labeled in Fig. S7a). The Zak phase of such 1D subsystem is quantized to either π or 0 owing to the mirror reflection about $y = 0$ plane. Moreover, due to the non-trivial Berry phases of Dirac nodal lines, the surface BZ is divided into two regions (inside or outside the two nodal lines) with different Zak phases. In Fig. S7b, we show the Zak phase sum ($\theta_1+\theta_2$) on a k_x line with $k_z = 0.2\pi/d$ (black line in Fig. S7c). Clearly, the Zak phase is π inside the two nodal lines while it is 0 outside, manifesting the non-trivial topology of the Dirac nodal lines. It indicates that a midgap surface state should exist in the momentum regime between two nodal lines when the crystal is truncated at inversion center. We calculate the Zak phase distribution in the whole surface BZ (Fig.

S7c). The surface BZ is divided into two regions, one with π Zak phase (magenta) and the other with 0 Zak phase (cyan).

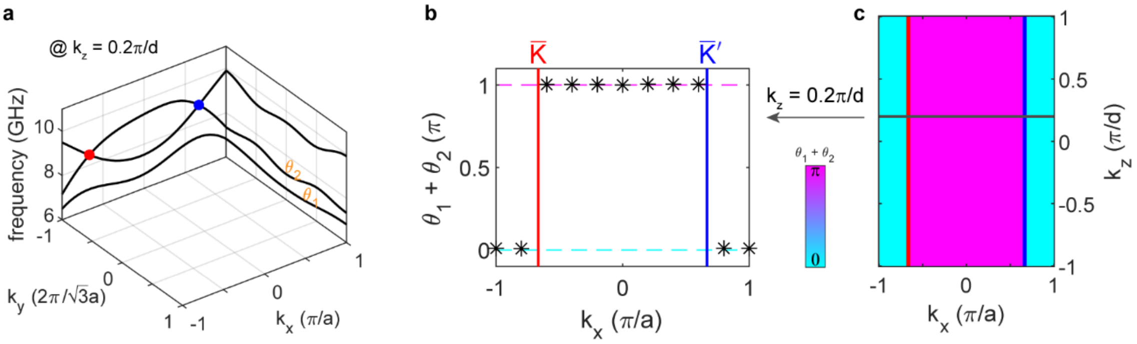

Fig. S7 **a** Bulk band structure on the plane of $k_z = 0.2\pi/d$. **b** Distribution of Zak phase sum ($\theta_1+\theta_2$) accumulated in the k_y direction, when $k_z = 0.2\pi/d$. **c** Zak phase distribution in the whole surface Brillouin zone.

Supplementary Note 4: Robust transport of antichiral surface state

Guaranteed by the non-trivial Zak phase distribution in the surface BZ (Fig. S7c), the topologically protected antichiral surface state is expected inside two nodal lines (Fig. 4). To demonstrate the robustness of the antichiral surface state, we perform full wave simulations with finite structures at 9.5 GHz. After obtaining the simulated 3D electric field, we perform FFT on the 3D field to extract the modes with positive k_z. According to our theory, the modes with positive k_z will propagate in the $+x$ direction.

First, we simulate the antichiral surface transmission on a boundary with metallic scatterer (Fig. S8a). For comparison, the upper surface is a flat surface while the lower surface is inserted with a metallic block as an obstacle. As shown in the intensity distribution (Fig. S8b), the surface states on two parallel surfaces are both excited to propagate rightward (positive k_z). Even though some of the EM waves are scattered into the bulk for the absence of a complete gap, most of the waves can wrap around the obstacle without backscattering.

In another case, antichiral transport on a bending surface is simulated in Fig. S8c. A metacrystal with a flat upper surface and an Ω-shaped lower surface is considered. From the intensity distribution (Fig. S8d), we can see that most of the waves can pass through the sharp corners and then propagate rightward.

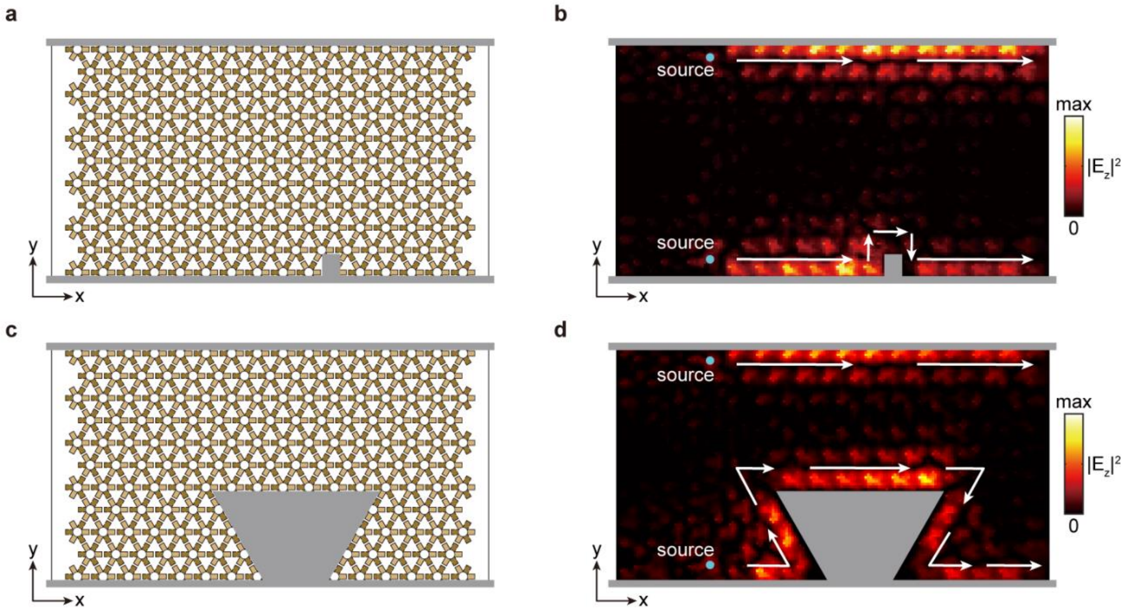

Fig. S8 **Simulated antichiral surface state with positive k_z. a** Schematic of the surface perturbation. The lower surface is perturbed by a metallic block, while the upper surface has no imperfection on the transport path. **b** Intensity profile of the excited surface states. **c** Schematic of the surface configuration with one straight path and one Ω-shaped path. **d** Antichiral surface transport on a flat surface and an Ω-shaped surface.

Supplementary Note 5: Reflection phase winding in the vicinity of Dirac point

As mentioned above, a topological antichiral surface always exists and connects the two nodal points. Previous work has studied the underlying mechanism of the ubiquitous existence of mid-gap surface states around the nodal point via surface reflection phase [Deng, et al., Light: Sci. Appl. 11:134 (2022)]. They found that the reflection phase will exhibit a 2π-winding in the vicinity of the nodal point and the nodal point serves as a singularity of the reflection phase. Consequently, the resonance condition, $\phi_{PC} + \phi_{PEC} = 2N\pi$, can always be satisfied and a surface state would always be found to pinned at the nodal point. Therefore, a drumhead/ribbon surface state ubiquitously exists on the surface between a Dirac nodal line semimetal and any gapped material. No matter the reflection phase of the gapped material, the resonance condition can always be satisfied, because of the 2π-winding near the nodal point. In other words, the existence of this kind of surface state is protected by bulk band topology (manifested as π Berry phase carried by the nodal point).

In a similar way, we calculate the reflection phase of our metacrystal, near the Dirac point. The top view (Fig. S9a) and the side view (Fig. S9b) of our setting to calculate the reflection phase are shown. A plane wave with *p* polarized is impinged onto the metacrystal. As an example, we focus on the reflection phase for $k_z = 0$ (i.e., consider the incident plane of xoy) and two values of k_x ($k_x = 0.6\pi/a$ and $k_x = 0.73\pi/a$, inside and

outside the nodal lines, see Figs. S9d & S9e). Obviously, a 2π-winding of reflection phase exists near the Dirac point. Therefore, the existence of our topological antichiral surface state is guaranteed by the 2π reflection phase winding near the nodal lines.

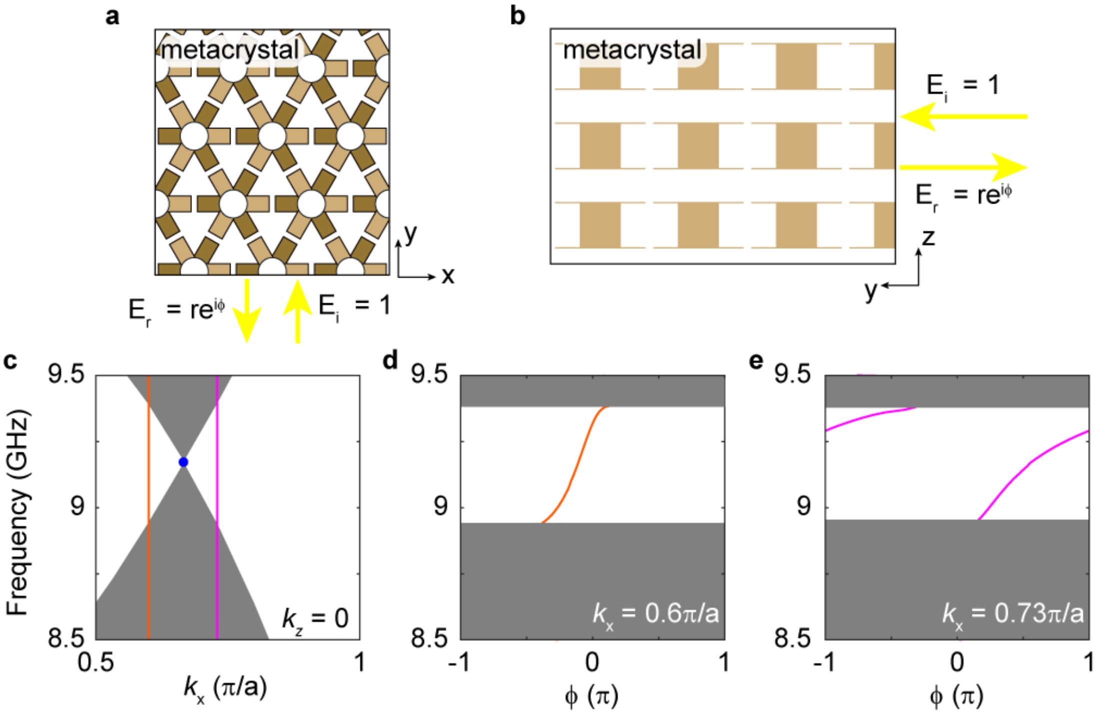

Fig. S9 **Reflection phase near Dirac point.** The top view (**a**) and the side view (**b**) of the setting to calculate the reflection phase. **c** Projected bulk band near the Dirac point with $k_z = 0$. **d-e** Reflection phase at $k_x = 0.6\pi/a$ and $k_x = 0.73\pi/a$.

Supplementary Note 6: Surface dispersion for different surface condition

To our knowledge, a nontrivial surface band should be supported inside or outside two projected nodal lines guaranteed by nontrivial Berry phases carried by two ANLs. Nevertheless, the surface dispersion would depend on specific surface condition of the metacrystal. In this section, we will show the surface dispersion in the situations with different surface termination.

<u>Surface dispersion for different types of surface truncation</u>

In Fig. S10, we define a parameter L to characterize the surface truncation. The red solid lines depict the practical boundaries (set as PEC) and the red dash lines depict the reference plane of L. L takes a positive value when the boundaries expand outward, whereas it takes a negative value when the boundaries shrunk inward. Without loss of generality, we focus on the cases with zero k_z in the following.

Figure S11 shows the surface spectra for different surface truncations. The surface dispersion will change its shape along with the changing of surface truncation. However, no matter how, the topologically protected surface states are always pinned at the two

Dirac cones (red and blue dots). The surface states locate inside the projected nodal lines when L range from 0 to $0.15a$, yet locate outside for L ranging from $-0.15a$ to 0.

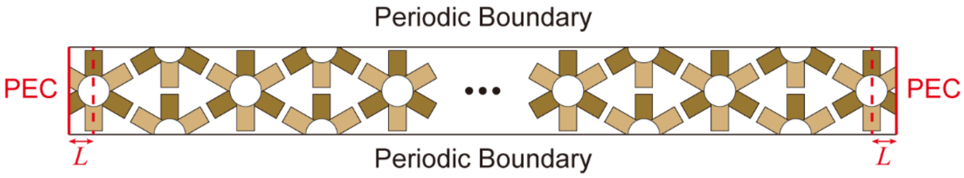

Fig. S10 Schematic of the surface termination. The red solid lines depict the crystal boundaries (set as PEC) while the red dash lines locate the reference planes of L.

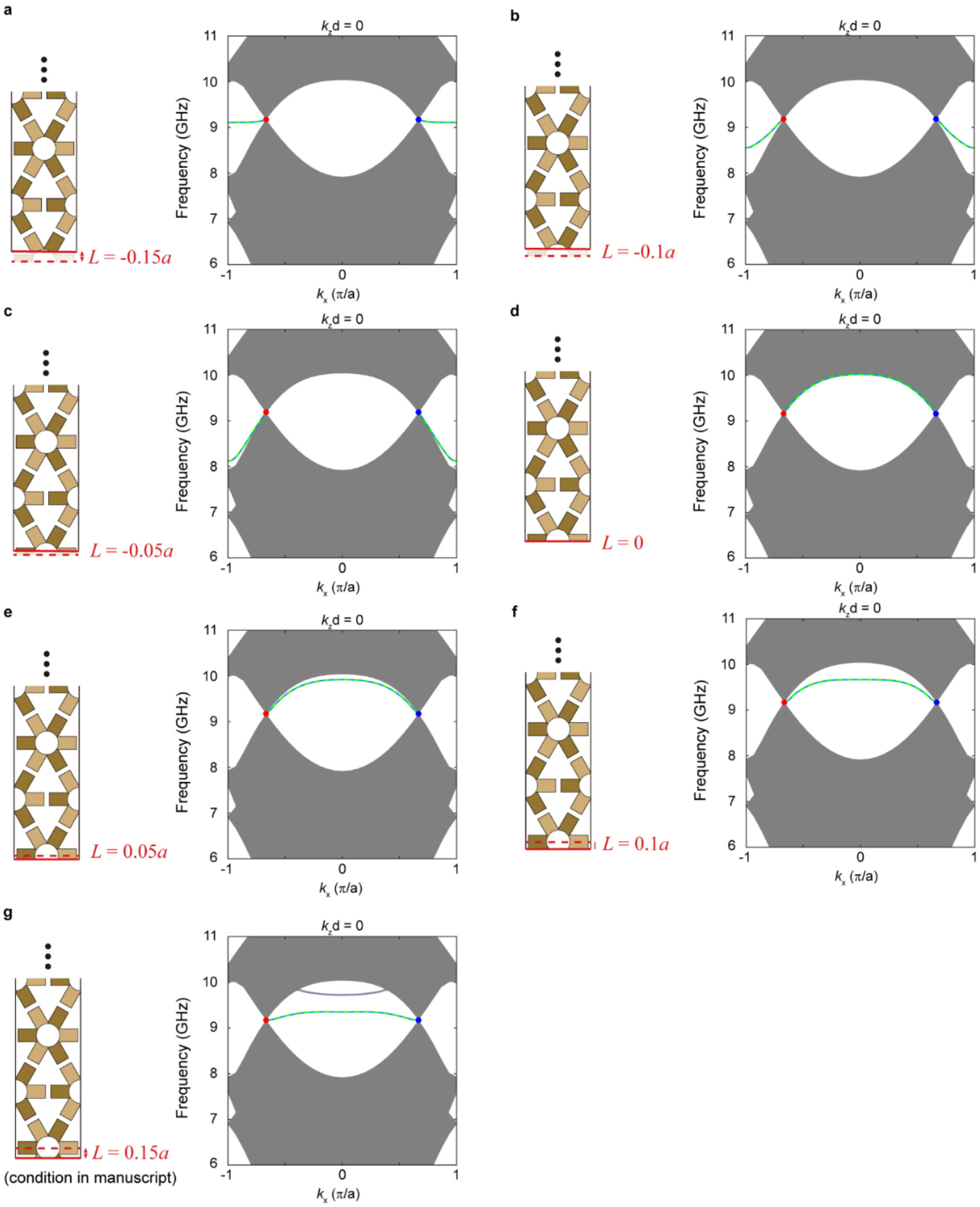

Fig. S11 **Surface dispersions for different surface truncation.** The red and blue dots

represent the Dirac points. The dark blue solid lines (green dashed lines) represent the surface dispersion for the upper (lower) surface, the purple line represents trivial surface mode.

Surface dispersion with an air gap between the crystal and PEC boundary

Besides the influence of surface truncation, the surface dispersion can be affected when an air gap exists between the metacrystal and the PEC boundary. Without loss of generality, we focus on the case with surface truncation $L = 0.15a$ and $k_z = 0.2\pi/d$. As shown in Fig. S12, the dispersion of the antichiral surface state can be significantly changed when introducing an air gap between the metacrystal and the PEC boundary. When an air gap is inserted, the topological surface dispersions (green and blue lines) change and become sagged. Besides, the trivial surface band (purple line) also changes. For example, when an air gap with thickness of 0.1 mm is inserted, the trivial band inside the Dirac points disappears but another band appears outside the Dirac points. The number of surface bands outside the Dirac points increases as the thickness of air gap further increases.

The reason for this influence is intuitive. For the case without an air gap (Fig. S12a), the outermost metallic particles of the crystal touch the PEC boundary directly. Consequently, all the outermost metallic particles are connected and have the same electric potential. This condition will be destroyed by inserting an air gap in between, because all the outermost metallic particles are isolated and can possess independent electric potential. As a result, the surface band dispersion has a drastic change.

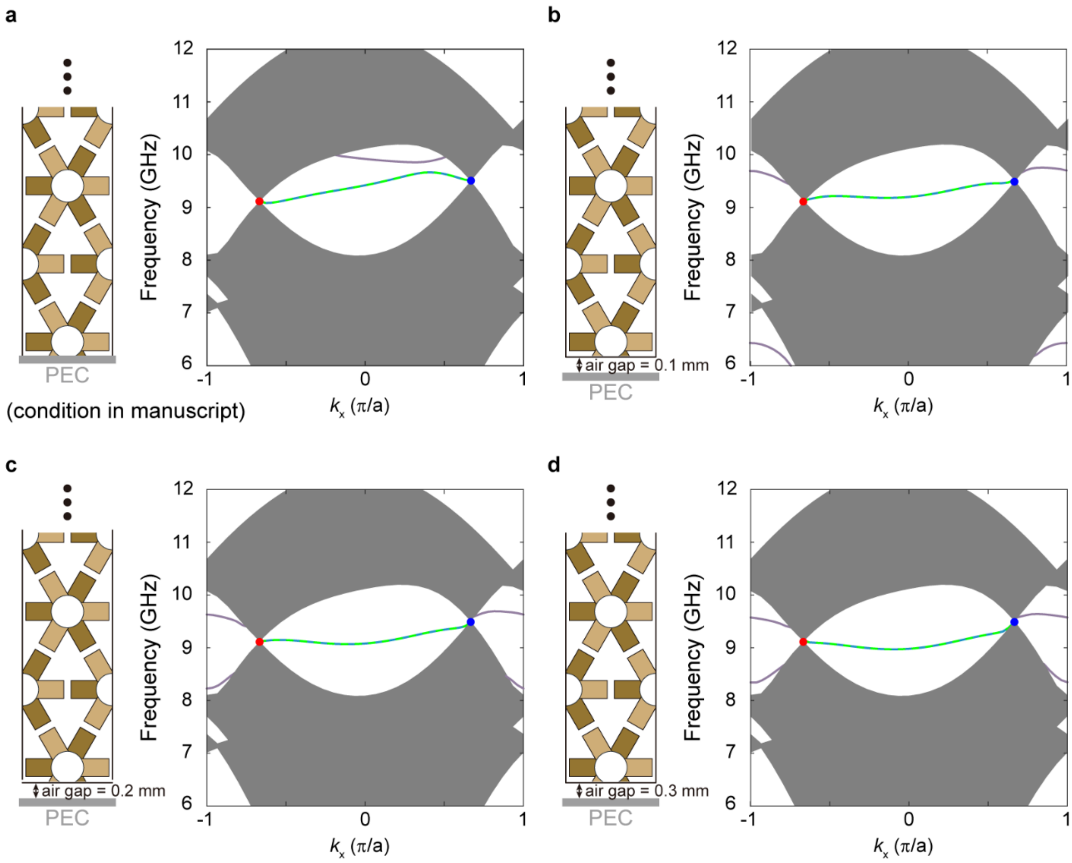

Fig. S12 **Surface dispersion with air gap between metacrystal and PEC boundary. a-d** Conditions for thickness of the air gap ranging from 0 to 0.3 mm.

Supplementary Note 7: Detailed experimental setup

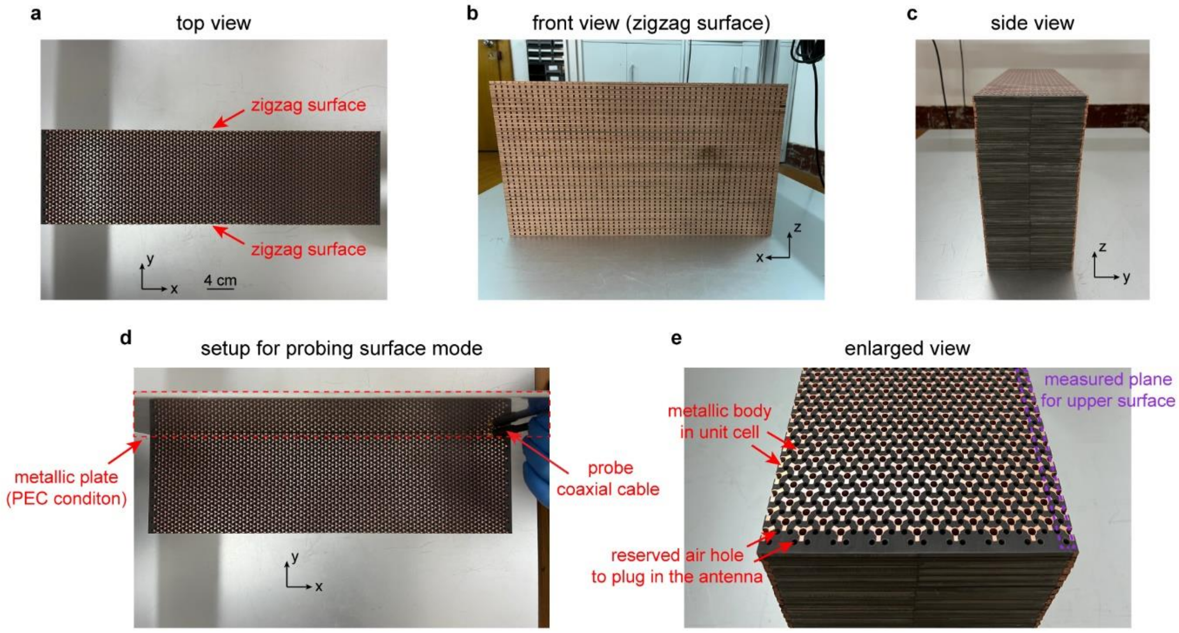

Fig. S13 **Detailed experiment setup. a** Top view of the sample. **b** The zigzag surface that supports the antichiral surface state. **c** Side view of the sample. **d** Experimental

setup for probing the antichiral surface state. A metallic plate is placed beside the sample to serve as a PEC. The probe antenna scans the electric field profile at each outmost reserved air hole (highlighted in Fig. S13e). **e** A enlarged view of the sample, showing the detailed metallic bodies in unit cell, the reserved air holes for plugging antennas and the surface truncation.

Supplementary Note 8: Directionality of the surface transport

To characterize the antichiral surface transport in our system, we define the directionality as the energy ratio between the field on the right of the source and the field on the left of the source, namely $\eta = 10\lg\left(\left|E_{z,\mathrm{Right}}\right|^2 / \left|E_{z,\mathrm{Left}}\right|^2\right)$. In this section, we illustrate in detail how we process the data. Figure S14a shows the surface electric field at 9.5 GHz, measured in experiment. A 6mm-long source antenna connecting to coaxial cable is plugged into the crystal at x = 240 mm. One can see a strong signal near the source cable. It is the excited transmission line mode formed between the source cable and the metallic boundary, covering up the field of antichiral waves. On the other hand, as we discuss in the main text, our system can be seen as a modified Haldane model with certain nonzero k_z. Thus, we perform a Fourier transform to extract modes with different k_z components first. As an example, figure S14b shows the extracted field with k_z = 0.2π/d. Then, we calculate the directionality of the extracted field. The dashed rectangles represent the regions to integrate the rightward propagating energy and leftward propagating energy, respectively. Finally, the directionality is obtained by the energy ratio. Notably, the region where the source antenna plugged in is not included in the integral.

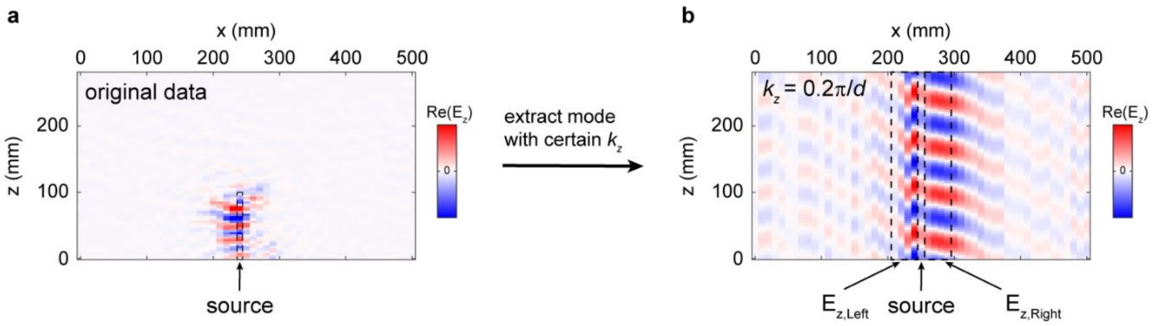

Fig. S14 Data processing for calculating the directionality of surface transport.

Supplementary Note 9: k_z-dependent antichiral surface transport

In this section, more results about the k_z-dependent antichiral surface transport are shown (results for k_z = 0.2π/d and k_z = 0.4π/d). The result for the upper surface (Figs. S15a-b) and the lower surface (Figs. S15c-d) are shown respectively. For the modes with positive k_z component, the directionality is almost positive in the frequency regime

(highlighted in yellow) corresponding to the tilted surface band. In addition, the electric fields measured in experiment are plotted, along with the simulated field profiles. In the experimental results, although the electric fields on the right of the source are obviously stronger than the ones on the left (indicating a unidirectional transport on the surface), the rightward propagating surface waves gradually decay in the *x* direction. The reason may be the imperfection of our sample. The sample is constructed by stacking PCBs in the z direction. The PCBs' out-of-flatness would lead to air gaps between neighboring boards and deteriorate the periodicity in the *z* direction. Then, surface waves with certain k_z could be easily scattered to the bulk states with other k_z, resulting in the attenuation of the antichiral surface waves.

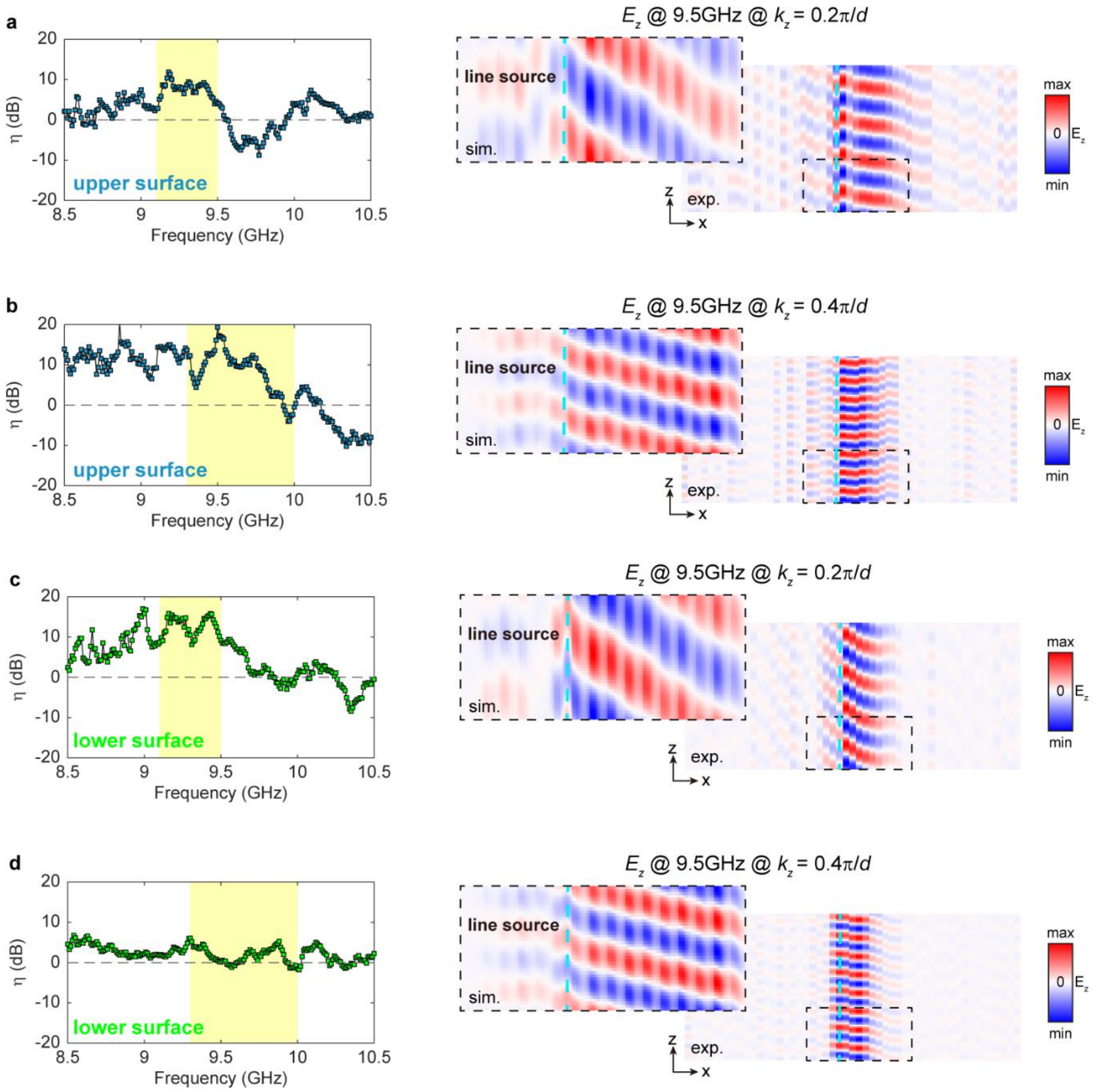

Fig. S15 Antichiral surface transport with different k_z component at different surfaces.
